# Intra-Articular Delivery of Nanoemulsified Curcumin Ameliorates Joint Degeneration in a Chemically Induced Model of Osteoarthritis

**DOI:** 10.3390/ijms262211212

**Published:** 2025-11-20

**Authors:** Kota Sri Naga Hridayanka, Shibsekhar Roy, Saikanth Varma, Navya Sree Boga, Archana Molangiri, Pradeep B. Patil, Myadara Srinivas, Asim K. Duttaroy, Sanjay Basak

**Affiliations:** 1 Molecular Biology Division, National Institute of Nutrition, Indian Council of Medical Research, Hyderabad 500007, India; hridayanka@gmail.com (K.S.N.H.); saikanthvarma50@gmail.com (S.V.); navyasreeboga.nice@gmail.com (N.S.B.); molangiri.archana@gmail.com (A.M.); 2 Department of Biochemistry, University College of Science, Osmania University, Hyderabad 500007, India; shibsekharroy@gmail.com; 3 Division of Animal Facility, National Institute of Nutrition, Indian Council of Medical Research, Hyderabad 500007, India; vetdrpradip@yahoo.com (P.B.P.); srinunin@gmail.com (M.S.); 4 Department of Nutrition, Institute of Basic Medical Sciences, Faculty of Medicine, University of Oslo, 0316 Oslo, Norway; a.k.duttaroy@medisin.uio.no

**Keywords:** curcumin nanoemulsion, osteoarthritis, inflammation, chondroprotective, intra-articular, macrophage recruitment

## Abstract

The pathogenesis of knee osteoarthritis (OA) is multifaceted and involves the complete joint microenvironment. Despite beneficial evidence of curcumin, the mechanistic insights of nanoemulsified curcumin (n-Cur) delivery to the knee-OA microenvironment are limited. The study aimed to establish localized delivery of curcumin nanoemulsion in the knee joint of OA rats and to examine detailed histopathological changes. n-Cur was prepared using a neutral dietary oil and a surfactant. Adult (5 mo) male SD rats were intra-articularly delivered 40 mg/mL of monoiodoacetate (MIA) to induce OA in the left knee and further treated with n-Cur (30 mg/mL). The effect of n-Cur on macrophage recruitment was evaluated using a co-culture model of CHON 001 and RAW 264.7 cells. In the MIA model, localized delivery of n-Cur significantly reduced knee joint edema and joint space narrowing in the target site. Curcumin ameliorated cartilage degeneration by reducing fibrillation, hypocellularity, and restoring matrix proteoglycan, as evidenced by histology. Reduced synovial inflammation displays the effect of curcumin on the synovium, possibly by lowering the recruitment of macrophages in chemoattractant-stimulated chondrocytes. Thus, curcumin nanoemulsion can act as a chondroprotective agent, modulating the OA microenvironment by reducing joint edema, synovial inflammation, and oxidative stress in the OA model.

## 1. Introduction

Knee osteoarthritis (OA) is a degenerative disease of the knee joints characterized by cartilage damage, synovial infiltration, and inflammation, which currently has no cure. Obesity, excessive mechanical stress on weight bearing joints, prior injuries, and age-related wear and tear increase the risk of developing osteoarthritis, thus emphasizing the need for early diagnosis [1]. Available pharmacological treatments and lifestyle modifications only provide symptomatic relief [2]. A major challenge to treatment options is the avascular nature of cartilage, which limits its ability to regenerate after wear and tear. Further, systemically administered drugs have shown limited therapeutic ability due to poor penetration. Compared to traditional drug delivery modes, intra-articular drug delivery is gaining traction due to its several benefits [3]. Despite this, intra-articular delivery of small molecules is prone to rapid clearance from the joint, resulting in less retention [4]. In this regard, complementary to site-specific delivery, nanoformulations are also known to increase solubility, enhance local bioavailability, and improve the compound’s overall absorption.

It is becoming increasingly evident that osteoarthritis is not just a cartilage degenerative disease but encompasses the complete joint microenvironment, including the subchondral bone, articular cavity, and the synovium. Pathological changes, including cartilage degeneration [5], joint edema [6], joint space narrowing, subchondral sclerosis [7], and synovial inflammation [8], occur in the knee joint during the progression of osteoarthritis. Despite the avascularity of cartilage, the vascular microenvironment in the surrounding tissues of the joint may contribute towards restorative pathways in OA.

In osteoarthritis pathogenesis, NFkB is a major regulator that modulates inflammation and degeneration [9]. NFkB is involved in the regulation of matrix-degrading enzymes and cartilage degeneration via the activation of various factors [10]. As an inflammatory modulator, NFkB plausibly exerts significant effects on the synovial membrane as well. Despite evidence in this regard [9], the majority of studies have explored the mechanistic changes in NF-κB only in relation to cartilage or chondrocytes [11,12]. The osteoarthritic synovium is characterized by the presence of fibroblasts in the lining layer, as well as macrophages and other infiltrated immune cells, including T and B lymphocytes [13]. Of these, macrophages are the prime mediators of inflammation, and macrophage-targeting is increasingly being reported for the alleviation of osteoarthritis [9,14]. Activated macrophages in the synovial membrane of the osteoarthritic microenvironment play a pivotal role in OA pathogenesis [15] and have been highlighted as possible therapeutic targets [16]. Recent evidences suggest cellular crosstalk between synovial cells and chondrocytes [17,18]. Despite these preliminary findings, studies investigating macrophage recruitment in OA are limited [19].

Recently, studies have demonstrated the chondroprotective properties of curcumin, a well-known polyphenol, in addition to its well-established anti-inflammatory effects [20,21]. The roles of curcumin in ameliorating osteoarthritis have been highlighted extensively [22]. Furthermore, formulating nanotherapeutic strategies for curcumin may enhance its bioactive effects and improve the chondroprotective effects [23]. Several preclinical studies have demonstrated the efficacy of various curcumin nanoformulations when administered intra-articularly, indicating anti-inflammatory and chondroprotective properties. Approaches such as curcumin-encapsulated microspheres [24], a core-brush nanoplatform containing curcumin [25], curcumin nanoparticles [26], or the synergistic delivery of curcumin and metformin nanoparticles [27] consistently demonstrate anti-inflammatory and anti-apoptotic effects in mitigating OA. For cartilage repair and regeneration, microenvironment-responsive nanomedicines show promising potential [28].

Despite reporting the efficacy and mechanistic evidence of curcumin on osteoarthritis pathogenesis [26,29,30], the mechanistic evidence of its effects on the OA microenvironment is limited. Macrophage recruitment is a central feature of synovial inflammation during osteoarthritis. The presence of infiltrated cells is explained by the activation state of macrophages (M1/M2), which constitute 43% of all infiltrated immune cells in the knee joint synovium during osteoarthritis [31]. Dysregulated cartilage has been shown to promote an inflammatory response in the synovium [8,32]. Synovial fibroblasts, inflammatory macrophages (M1), immune regulatory macrophages (M2), and dendritic cells abundantly constitute the synovium and produce major pro-inflammatory cytokines, including IL1β [33]. Few studies have investigated the impact of curcumin on synovial inflammation [26,29,30]; however, the roles of infiltrated macrophages in relation to chondrocytes have not been reported with nanoemulsified curcumin.

This study aimed to investigate the effects of intra-articularly delivered curcumin nanoemulsion on the mechanisms occurring in the joint microenvironment during osteoarthritis. The monoiodoacetate (MIA)-induced model of osteoarthritis closely mimics human osteoarthritis in terms of biochemical, histological, and inflammatory changes [34]. Mimicking the osteoarthritis microenvironment, this study employed a co-culture model to evaluate the chemotactic effect of IL1β-stimulated chondrocyte-derived factors on the migration of macrophages. Curcumin is a lipophilic molecule with poor solubility in water. The use of the nanoemulsion form warrants better solubilization of curcumin in an oil-in-water approach. In addition to providing better uptake of oil-soluble components such as curcumin, nanoemulsions also provide increased retention time in the joint space suggesting extended release over native curcumin [35]. Curcumin nanoemulsion was prepared in a micellar form using a dietary oil formulated with curcuminoids.

This study provides the first report on the effects of nanoemulsified curcumin on OA-induced cartilage fibrillation in rats and inflammatory modulating activities in the microenvironment associated with osteoarthritis.

## 2. Results

### 2.1. Curcumin Nanoemulsion Reduced MIA-Induced Knee Joint Edema and Joint Space Narrowing in Osteoarthritic Rats

**Figure 1:**
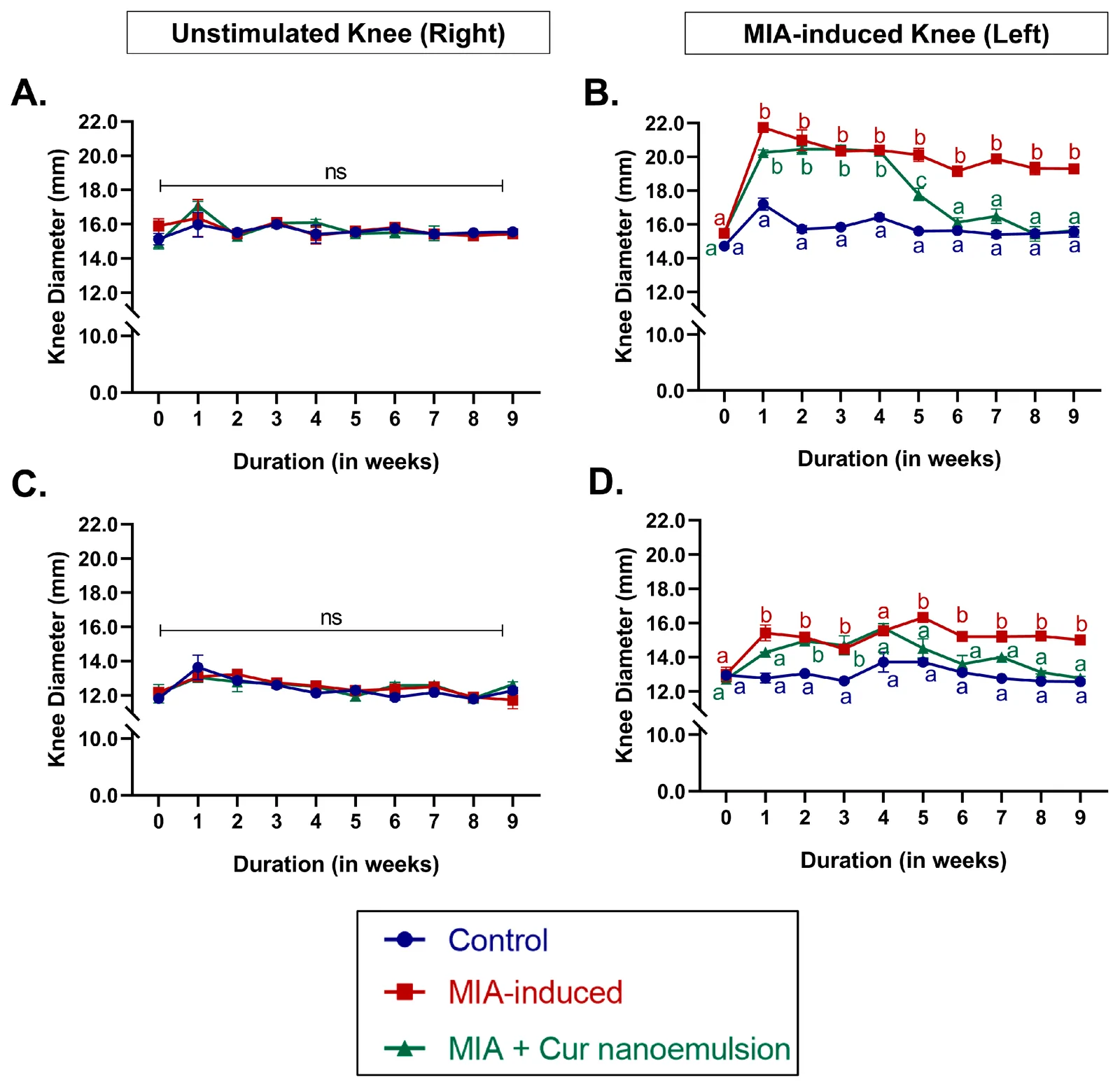
The effect of curcumin nanoemulsion on joint edema in osteoarthritis rats. Knee diameter in rats was measured at different time points using digital vernier calipers to monitor joint edema. (**A**,**B**) Anteroposterior knee diameter (in mm) of an unstimulated (**A**) and MIA-induced knee (**B**); (**C**,**D**) Mediolateral knee diameter (in mm) of an unstimulated (**C**) and MIA-induced knee (**D**), respectively. Data were analyzed using a repeated-measures mixed-effects model with Tukey’s multiple comparison tests and represented as mean ± SEM (*n* = 3/group). ns indicates no significant difference and values with unlike letters were considered significant with *p* < 0.05 vs. control.

The formulated curcumin nanoemulsion was characterized with a size of 196.94 ± 12.65 nm, a negative zeta potential of 33.30 ± 0.70 mV, and a PDI (%) of 29.85 ± 1.35 (Table S1). Knee joint edema is a key characteristic symptom of osteoarthritis. To understand its effect on joint edema in an MIA-induced osteoarthritis model, joint diameter was measured. One week after the MIA injection, both anteroposterior and mediolateral joint diameter of the affected knee increased significantly and remained above the baseline throughout the study duration, indicating OA-related edema (Figure S1). Intra-articular injection of curcumin nanoemulsion for four weeks significantly reduced edema of the knee joint, with joint diameter being comparable to that of the control at the end of the study duration (Figure 1B,D). Joint edema and the subsequent effect of nanocurcumin were site-specific and did not alter anteroposterior or mediolateral knee joint diameter of the unstimulated knee (Figure 1A,C).

**Figure 2:**
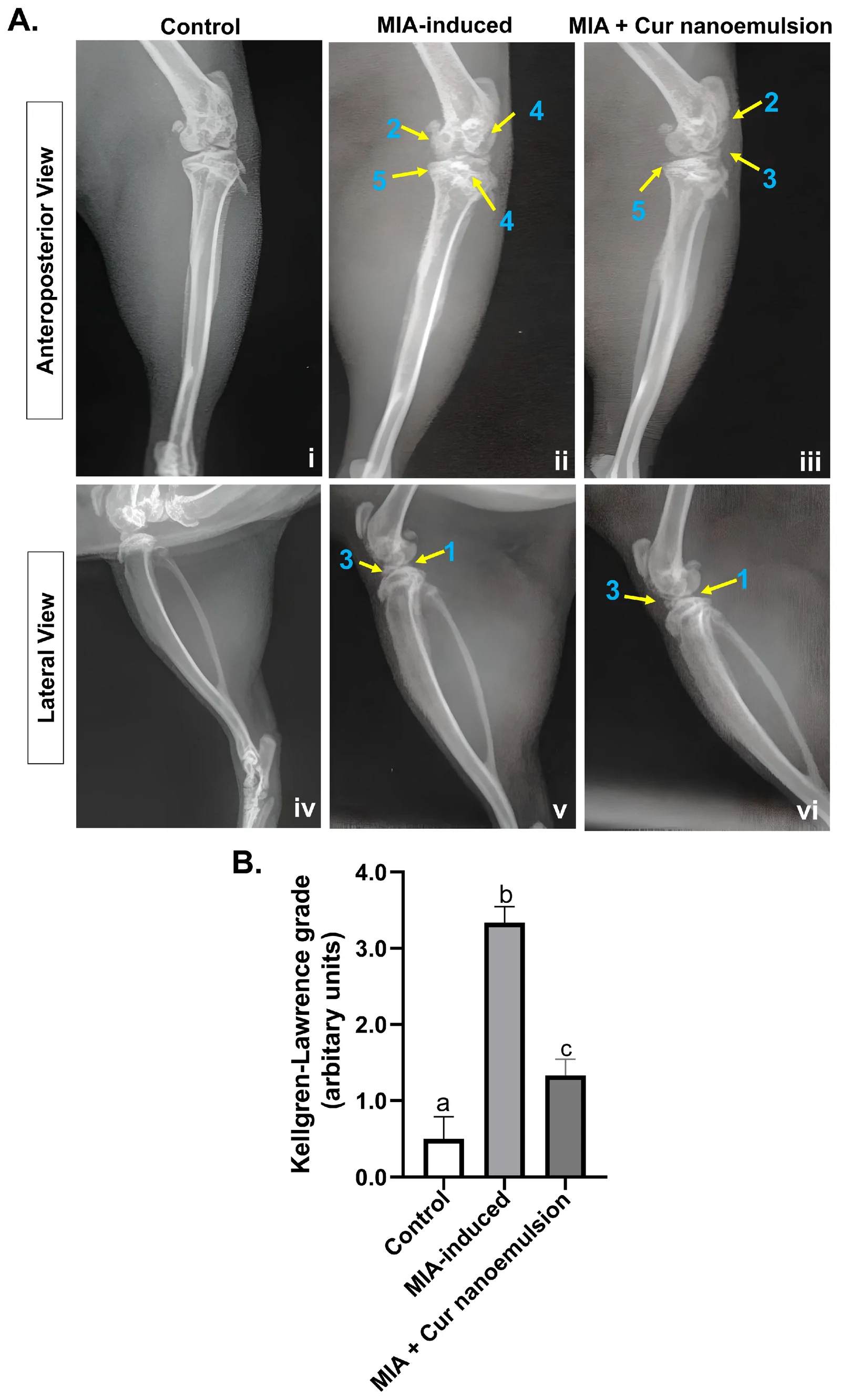
X-ray radiographs of rat knee joints induced with monoiodoacetate and subsequently treated with curcumin nanoemulsion. (**A**) X-ray radiographs of the knee joint in anteroposterior (**i**–**iii**) and lateral (**iv**–**vi**) planes. (**i**,**iv**)—The control rat showed a well-defined joint space with uniform and smooth bone contours. (**ii**,**v**)—MIA-induced rats revealed narrowing of the joint space (1), roughened surfaces of bicondyles (2), increased opacity indicating joint effusion (3), subchondral sclerosis (4), and possible osteophytes (5). (**iii**,**vi**)—Treatment with curcumin nanoemulsion restored joint space narrowing (1) and reduced opacity (3), despite slight surface irregularity (2) and possible osteophytic lipping (5). (**B**)—Mean osteoarthritis grade using the Kellgren–Lawrence classification. Data are presented as mean ± SEM (*n* = 3/group) and analyzed using the non-parametric Kruskal–Wallis test followed by Dunn’s test for multiple comparisons. Unlike letters represent significance at *p* < 0.05 compared to the control.

Knee joints of control rats were observed to have smooth bone contours with a defined joint space (Figure 2(Ai,Aiv)). The most prominent MIA-induced structural alterations of the knee joint included joint effusion as indicated by increased opacity (Figure 2(Av)), articular cavity erosion indicated by roughened surfaces of bicondyles, subchondral bone sclerosis, and possible osteophytes (Figure 2(Aii)). Intra-articular injections of curcumin nanoemulsion led to substantial improvement in joint space narrowing and joint effusion (Figure 2(Aiii,Avi)). Slight surface irregularities were observed in addition to possible osteophytic lipping (Figure 2(Aiii)). Curcumin nanoemulsion significantly mitigated MIA-induced osteoarthritis [MIA-induced vs. Cur nanoemulsion: 3.33 ± 0.21 vs. 1.33 ± 0.21 (a.u.), arbitrary units, *n* = 3/group] by reducing effusion and joint space narrowing (Figure 2B) based on the Kellgren–Lawrence classification system for osteoarthritis grading of knee joints (Table S2).

### 2.2. Curcumin Nanoemulsion Alleviated MIA-Induced Cartilage Fibrillation, Reduced Systemic Oxidative Stress, and Synovial Inflammation in the Knee Joint

**Figure 3:**
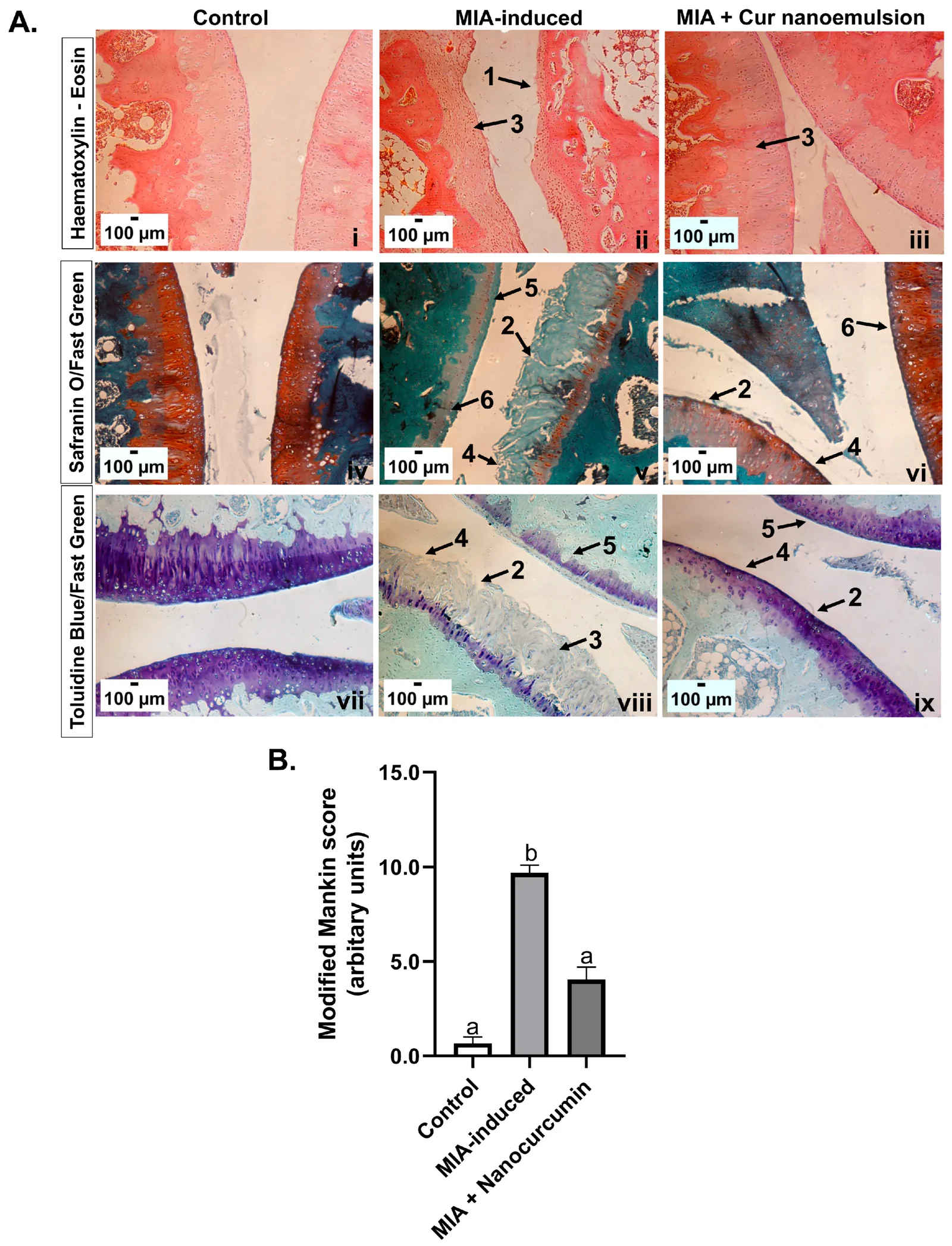
Histopathological assessment using different staining methods and quantitative scoring of the knee joint in MIA-induced osteoarthritis rats treated with curcumin nanoemulsion. (**A**) Histology sections of the femorotibial joint in rats stained with (**i**–**iii**) Haematoxylin-Eosin, (**iv**–**vi**) safranin O/fast green, and (**vii**–**ix**) toluidine blue/fast green for control, MIA-induced rats treated with or without nanoemulsion (magnification 10×, scale bar 100 μm). Control rats (**i**,**iv**,**vii**) exhibited a smooth cartilage surface and an intact tidemark, with normal cellularity and staining. MIA-induced rats (**ii**,**v**,**viii**) showed distinct cartilage erosion (1), deep fibrillation (2), loss of tidemark (3), depleted staining of matrix proteoglycans (4), matrix atrophy (5), and hypocellularity (6). Curcumin nanoemulsion (**iii**,**vi**,**ix**) increased matrix proteoglycans staining (4) displayed mild matrix atrophy (5), reduced fibrillation (2), reduced hypocellularity (6) and restored tidemark integrity (3). (**B**) Quantification of the osteoarthritic knee joints using modified Mankin scoring. Data were analyzed using the non-parametric Kruskal–Wallis test with Dunn’s multiple comparisons and represented as mean ± SEM (*n* = 3), with unlike letters denoting significance at *p* < 0.05 compared to the control.

Osteoarthritic histology exhibited deep fibrillation of cartilage, distinct erosion, and loss of tidemark in the femorotibial joint (Figure 3(Aii,Av,Aviii)). In contrast, normal cellularity, surface, and intact tidemark were observed in the control (Figure 3(Ai,Aiv,Avii)). Safranin O/fast green and toluidine blue staining revealed depletion of matrix proteoglycans, matrix atrophy, and loss of chondrocytes compared to control. In contrast, curcumin nanoemulsion restored matrix proteoglycans, reduced cartilage erosion, fibrillation, and matrix atrophy (Figure 3(Aiii,Avi,Aix)). Histological findings were scored based on the modified Mankin score (Table S3). Further, nanoemulsion significantly decreased modified Mankin score (MIA-induced vs. Cur nanoemulsion: 9.70 ± 0.40 vs. 4.05 ± 0.66, *n* = 3/group) by reducing chondrocyte loss and restoring tidemark (Figure 3B).

**Figure 4:**
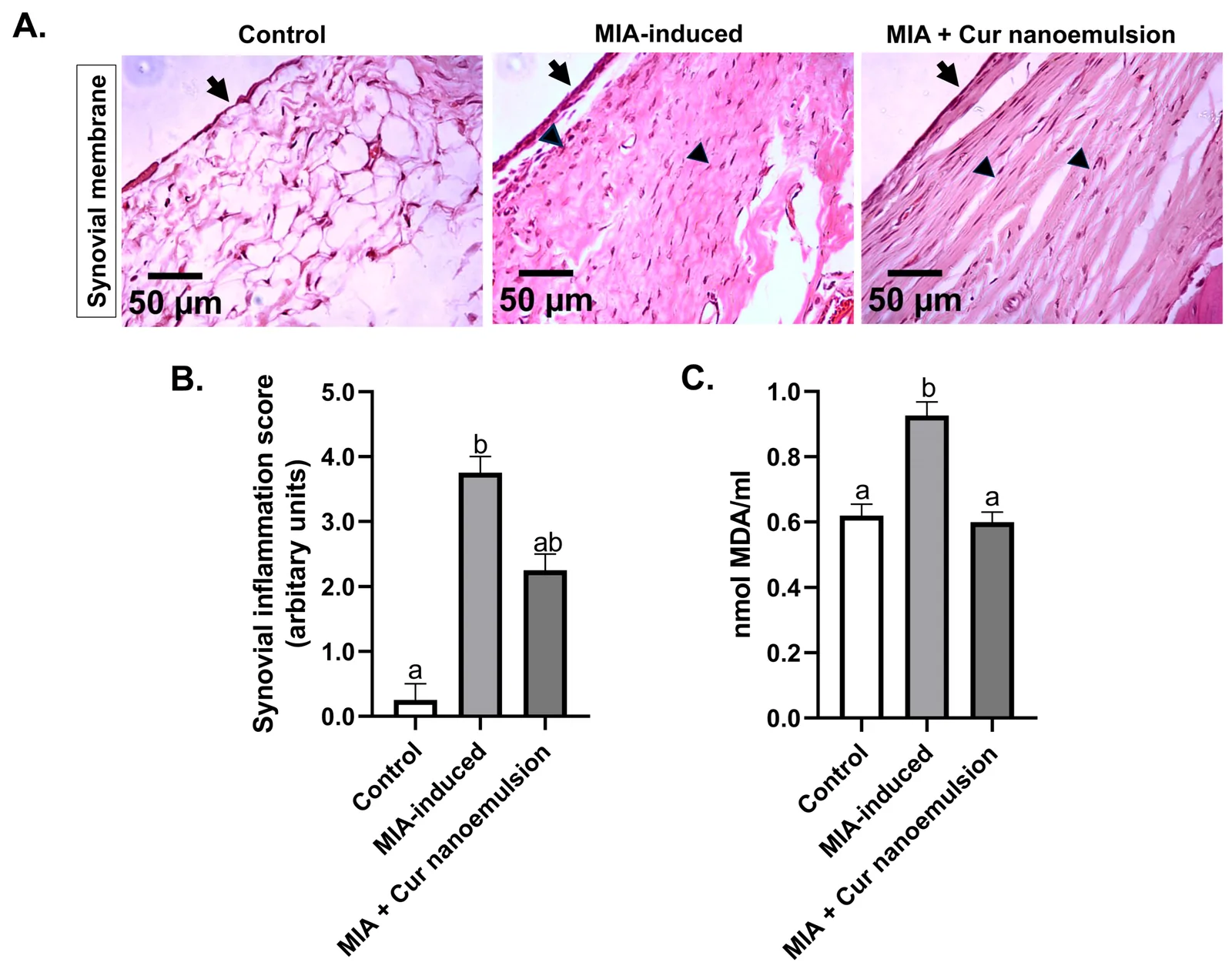
Effect of curcumin nanoemulsion on synovial inflammation and oxidative stress in MIA-induced OA rats. (**A**) Representative histology sections of the synovial membrane in the knee joint of rats stained with Haematoxylin-Eosin (40×, scale bar 50 μm). Black arrows highlight the synovial lining, and the pointed triangle indicates infiltration of inflammatory cells. (**B**) Quantification of synovial inflammation. (**C**) Systemic oxidative stress measured by TBARS assay. Data were analyzed using non-parametric Kruskal–Wallis and Dunn’s multiple comparisons and represented as mean ± SEM (*n* = 3/group), with unlike letters denoting significance at *p* < 0.05 compared to the control.

In addition, H&E staining of the synovial membrane in osteoarthritic rats revealed an increased number of cell layers in the synovial lining (Table S4) which significantly reduced post treatment with nanoemulsion (Figure 4A,B). Nanoemulsion effectively decreased infiltration of inflammatory cells and proliferation of the sub-synovial tissue compared to OA (MIA-induced vs. Cur nanoemulsion: 3.75 ± 0.25 vs. 2.25 ± 0.25 a.u., n = 3/group). In addition, upregulated MDA levels in OA rats were significantly reduced with curcumin nanoemulsion injections (MIA-induced vs. Cur nanoemulsion: 0.92 ± 0.04 vs. 0.59 ± 0.03 a.u., *n* = 3/group), implicating a decrease in systemic oxidative stress (Figure 4C).

### 2.3. Curcumin Nanoemulsion Reduced In Vitro Macrophage Migration Towards Chondrocytes in the Presence of IL1β

**Figure 5:**
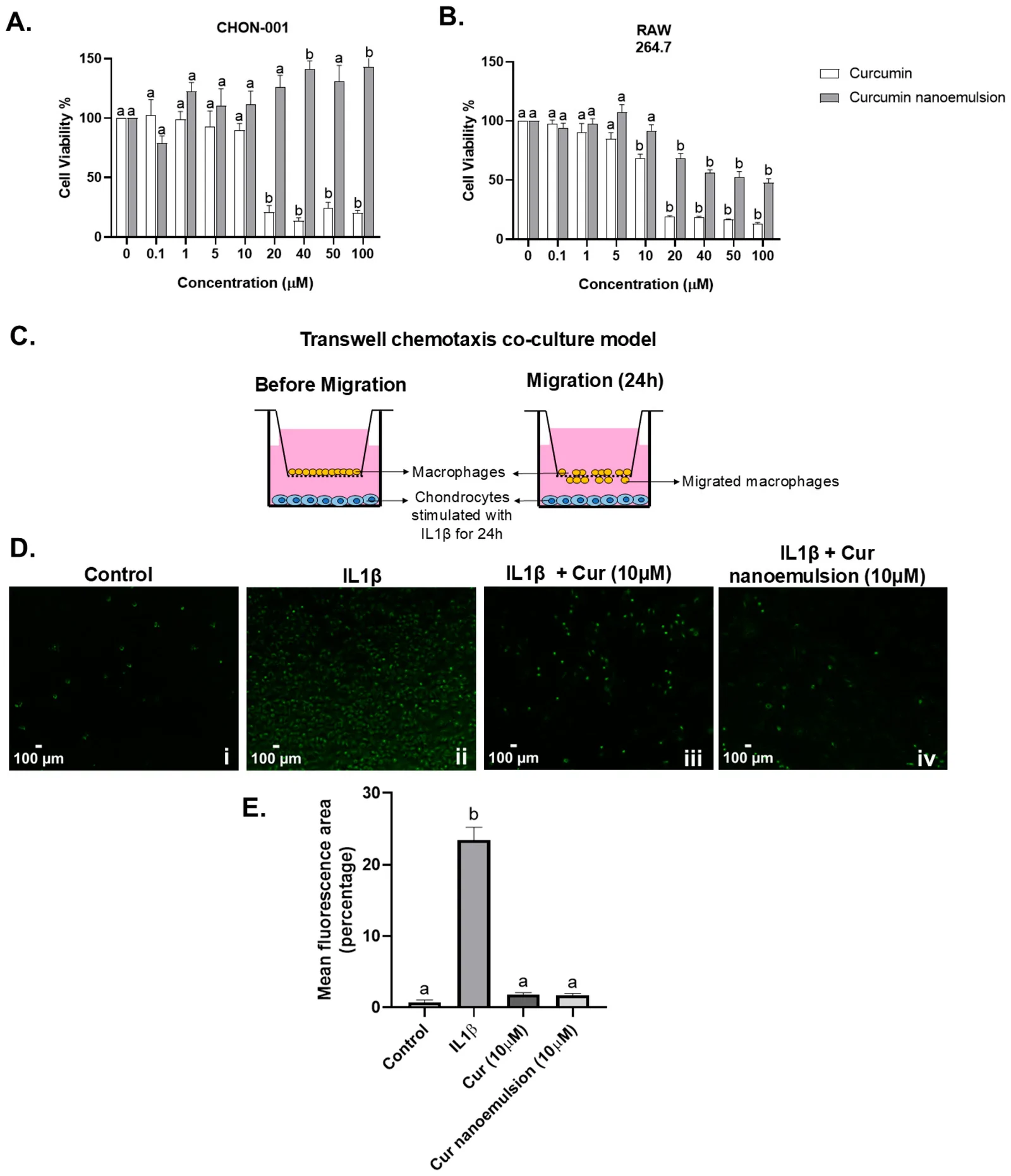
Effect of curcumin nanoemulsion on cell viability and chemotactic migration of macrophages towards IL1β-induced chondrocytes. (**A**,**B**) Cell viability by CHON-001 and RAW 264.7 cells, respectively, exposed to different concentrations of curcumin and nanoemulsion (0.1–100 μM) for 24 h. Cell viability was evaluated using the MTT assay and calculated by comparing optical density values between the control and treatment groups after normalizing them with blank values, and expressed as a percentage of the control. Each value is represented as mean ± SEM (*n* = 8). (**C**) Transwell co-culture model. (**D**) Representative images of calcein AM (green)-stained macrophages on the underside of 8 μm insert after 24 h of co-culture. Chondrocytes were cultured in the lower chamber and exposed to stimulus, while macrophages were incubated in the insert with plain assay media. Images (magnification, 10×; scale bar, 100 μm) represent macrophage migration towards (**i**) unstimulated chondrocytes, (**ii**) IL1β-induced chondrocytes, (**iii**) IL1β-induced chondrocytes treated with curcumin (10 μM), and (**iv**) IL1β-induced chondrocytes treated with nanoemulsion (10 μM). (**E**) Quantification of migrated cells using mean fluorescence intensity per unit area. Data were analyzed using one-way ANOVA with Tukey’s multiple comparisons, and *p* < 0.05 was considered statistically significant compared to the control. Unlike letters denote significant differences.

Synovial inflammation and associated macrophage infiltration are key to the pathogenesis of osteoarthritis. To mimic the osteoarthritis microenvironment in vitro, we employed an indirect transwell co-culture system to check macrophage response to the release of chemokines and soluble factors from chondrocytes. Two cell lines, chondrocytes (CHON-001) and macrophages (RAW264.7) were employed. A dose–response assay was performed using the MTT assay to evaluate cell viability when cells were exposed to different concentrations (0.1 to 100 μM) of curcumin or curcumin nanoemulsion (Figure 5A,B). Compared to native curcumin, nanoemulsion maintained cell viability even at high concentrations in both chondrocytes and macrophages. Further, in chondrocytes, curcumin nanoemulsion treatment retains viable cells even at 20–100 μM concentrations. For all subsequent experiments, concentrations of 5 or 10 μM were used for both curcumin and the nanoemulsion.

To assess the potential of curcumin nanoemulsion in mitigating the macrophage response and migration towards inflamed chondrocytes, a chemotactic gradient was established using IL1β. Chondrocytes were added to the lower chamber, while unstimulated macrophages were incubated on inserts. The co-culture setup prevented direct cell-to-cell contact and facilitated only the transfer of soluble factors, including chemokines, while also aiding the migration of macrophages in the presence of a strong chemotactic signal from chondrocytes. Chondrocytes exposed to IL1β promoted chemotactic migration of macrophages (Figure 5(Dii)), which was reduced significantly upon treatment with curcumin. Both curcumin and nanoemulsion reduced macrophage migration despite the presence of IL1β (Figure 5D). Basal migration of macrophages towards unstimulated chondrocytes was present but minimal.

### 2.4. Modulation of Pro-Inflammatory Mediators and Matrix Regulators in Chondrocytes Exposed to Curcumin Nanoemulsion

We further analyzed the effect of curcumin nanoemulsion on the expression of pro-inflammatory modulators and matrix regulators in chondrocytes. Foremost, stability values for a panel of endogenously expressed genes (Table S5) were assessed, and TBP was chosen as an endogenous control due to its highest stability for the given experimental conditions in CHON-001 cells (Table S6). *NFKB* expression was upregulated significantly upon IL1β stimulation (Table 1), while nanoemulsion downregulated the expression of *NFKB*. In contrast, *NFKBIB* was upregulated upon exposure to nanoemulsion (Table 1). *TGFB1* was significantly downregulated by ~2-fold upon IL1β stimulation, while curcumin nanoemulsion restored *TGFB1* expression. Upregulated levels of *TNFA* by IL1β were significantly reduced in cells exposed to nanoemulsion. Bone morphogenetic protein 2 (*BMP 2*) was upregulated following IL1β stimulation. It was further upregulated by curcumin and curcumin nanoemulsion despite no significant difference between curcumin and IL1β. Both curcumin and nanoemulsion upregulated *SMAD1*; only nanoemulsion significantly downregulated *SMAD5* expression. Expression of osteonectin (*SPARC*) was significantly downregulated by IL1β, which was further reduced in cells exposed to nanoemulsion. The relative mRNA expression of *MMP2* did not change significantly with curcumin and nanoemulsion; however, curcumin, but not the nanoemulsion, downregulated *MMP-9*. Significant upregulation in expression of *MMP14* and *TIMP1* was observed in curcumin and nanoemulsion-exposed cells.

### 2.5. Curcumin Nanoemulsion Lowered IL1β-Induced Expression of NFκB in Macrophages (RAW264.7)

**Figure 6:**
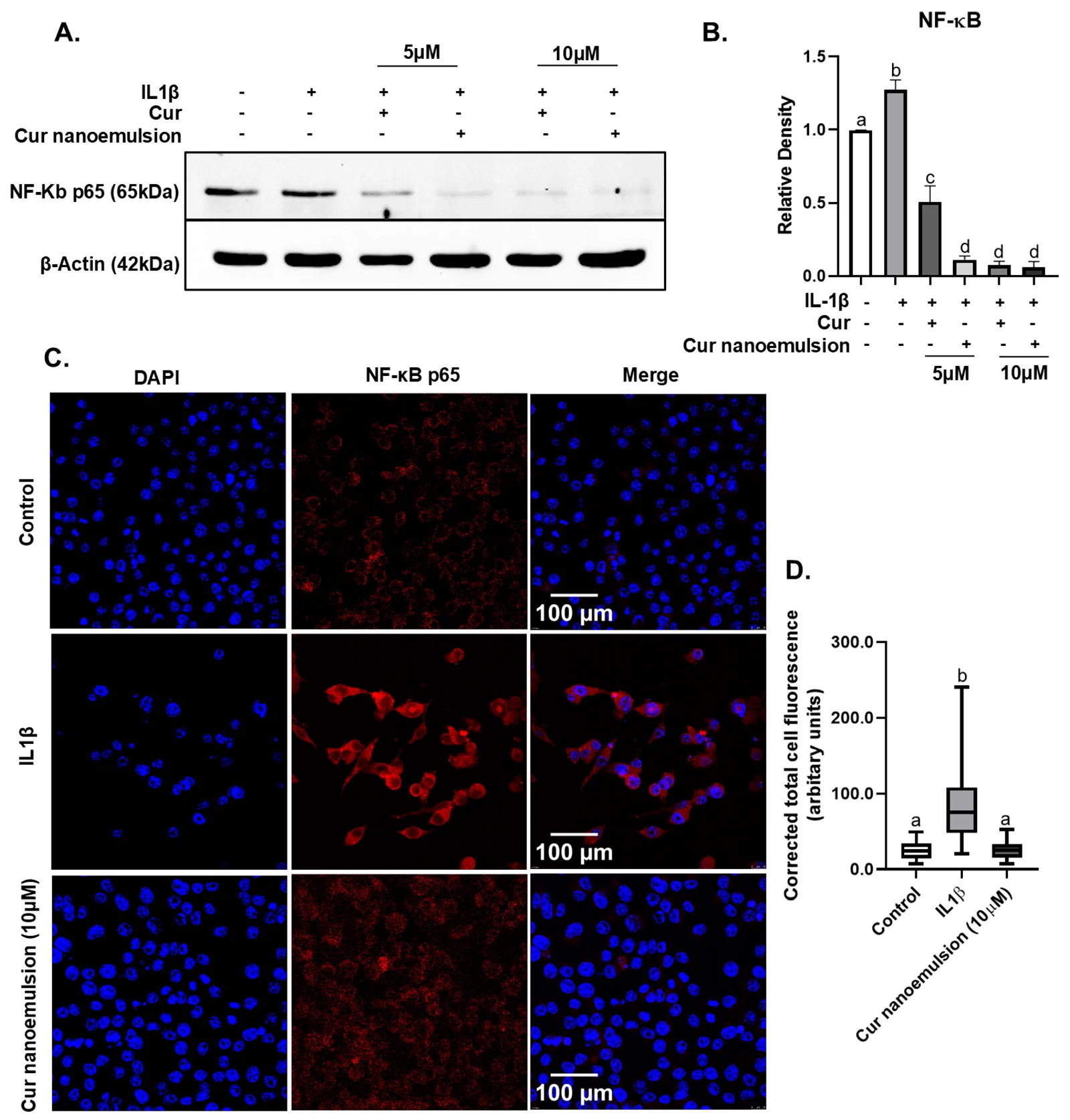
Expression of inflammatory mediator NF-κB in RAW 264.7 macrophages following IL1β stimulation in the presence of curcumin/nanocurcumin (5–10 μM). (**A**) Immunoblot of NF-κB and (**B**) its relative protein expression normalized with β-actin (mean ± SEM). (**C**) Nuclear and cytosolic expression of NF-κB in RAW 264.7 macrophages using immunocytochemistry after treating with IL1β and curcumin nanoemulsion (10 μM). Cells were stained with Alexa Fluor 594-tagged NF-κB (red) and DAPI (blue) to stain nuclei with original magnification of 40×; scale bar 100 μm. Images were captured using a confocal laser scanning microscope with an oil immersion objective (TCS SP5, Leica Microsystems, Germany). (**D**) Corrected total cell fluorescence (CTCF) was calculated, analyzed statistically and expressed in arbitrary units [CTCF = integrated density—(area of selected cell × mean fluorescence of background readings)]. Data were analyzed using one-way ANOVA with post hoc Tukey’s multiple comparison test, with *p* < 0.05 considered significant, and results depicted using different letters.

Macrophages were stimulated with IL1β to mimic osteoarthritis-related inflammatory responses in vitro and then treated with curcumin or nanoemulsion. In macrophages, both curcumin and curcumin nanoemulsion displayed a dose-dependent decrease in the expression of NFκB (Figure 6A). However, nanoemulsion at 5 μM concentration was significantly more effective than native curcumin in reducing the expression (Figure 6B). Further, we evaluated the localization of NFκB in macrophages using immunocytochemistry. For this, cells were treated with either IL1β or a combination of IL1β and curcumin nanoemulsion (10 μM). An upregulation in overall cellular expression of NFκB (Figure 6C) in cells exposed to IL1β (10 ng/mL) was observed. Curcumin nanoemulsion (10 μM) was found to significantly downregulate both nuclear and cytoplasmic expression of NFκB (Figure 6D).

## 3. Discussion

This study demonstrated that targeted delivery of curcumin nanoemulsion by intra-articular administration ameliorates osteoarthritis pathogenesis in vivo by modulating joint space narrowing, cartilage degeneration, and synovial inflammation. This study provides novel evidence that nanoemulsified curcumin reduces osteoarthritis-induced cartilage fibrillation in rats and mitigates macrophage recruitment towards chemoattractant-stimulated chondrocytes, suggesting its inflammatory modulating activities in the microenvironment associated with osteoarthritis. Curcumin nanoemulsion attenuated structural alterations of osteoarthritis, including joint edema, cartilage erosion, and subchondral sclerosis, by reducing inflammation and oxidative stress in the joint microenvironment.

Extensive thickening of the synovial membrane lining and infiltration of inflammatory cells were observed in OA rats in this study, suggesting synovial inflammation [36]. In this study, curcumin nanoemulsion reduced the thickening of synovial lining despite reduced but persistent infiltration. Persistent infiltration can be attributed to the presence of macrophages in the synovium [31]. The synovial membrane lines the inner joint capsule and consists of a lining cell layer, an intima layer (consisting of synovial fibroblasts and macrophages) and subintima layer (connective tissue in continuation with adipose tissue). As observed in the control group, adipose-type synovium is noted as a morpho-functional unit in conjunction with the infrapatellar fat pad in the knee joint [37]. Immune cell infiltration, majorly macrophages in the subintima layer observed in the MIA-induced group, may cause structural remodeling of the joint adipose tissue resulting in fibrosis or altered adipocyte morphology [38,39].

Similarly to the synovium, the subchondral bone and the joint cavity contribute to different mechanisms in promoting osteoarthritis pathogenesis. Significant changes in the subchondral bone, increased joint edema, and effusion observed in this study might contribute towards cartilage degeneration [7,40]. The reduction in edema and effusion in the joint after treatment with nanoemulsion, as also reported previously [26,27], underscores its function in mitigating osteoarthritis-related inflammation. Effusion was characterized qualitatively based on opacity as reported previously [41].

Further, joint space narrowing and possible osteophytes were observed in the OA model. Joint space narrowing, a characteristic feature of the MIA model, is associated with both pain and inflammation [42]. The reduction in joint space narrowing due to curcumin nanoemulsion delivery suggests its beneficial effects in alleviating pain [43,44]. In the OA model, rough surfaces of the bicondyles indicated cartilage erosion, which was lessened with curcumin nanoemulsion, demonstrating its chondroprotective role. Additionally, synovial inflammation and structural changes are closely linked to pain responses in osteoarthritis [42]. Transcriptome analysis of primary articular chondrocytes derived from different pathological grades of osteoarthritis revealed significant alterations in gene expression responsible for immune response, cell adhesion, and extracellular matrix production, thus validating the involvement of the complete joint environment [45]. The inflammatory microenvironment of osteoarthritis significantly contributes to oxidative stress [46]. Consistent with earlier findings [44,47], lower MDA levels, which assess oxidative damage in the affected tissues, indicate curcumin’s role in restoring oxidative balance.

Enhanced migration of macrophages toward OA-like chondrocytes observed in this study highlights the role of both IL1β as a chemoattractant and soluble factors secreted by the chondrocytes that facilitate crosstalk with macrophages [48,49]. Conversely, the curcumin nanoemulsion significantly decreased the chemotactic migration of macrophages, emphasizing the role of the chondrocyte secretome in macrophage recruitment. These findings offer new mechanistic insights into how curcumin affects chondrocytes in an inflammatory environment, contributing to macrophage recruitment and synovial inflammation.

In line with previous reports [5,50], this work also observed that monoiodoacetate led to a prominent degeneration of the articular cartilage, accompanied by erosion, fibrillation, chondrocyte loss, and reduced proteoglycan staining. This can be explained by alterations in the expression of matrix metalloproteinases [51] and TGFβ [52,53]. In this study, curcumin nanoemulsion was found to reduce cartilage erosion, fibrillation, and hypocellularity. These structural alterations can be attributed to curcumin’s potential impact on the extracellular matrix and cartilage homeostasis [54]. In an explant OA model, curcumin has been previously reported to inhibit the degradation of the extracellular matrix and the release of glycosaminoglycans [55].

Previous studies on curcumin nanoemulsion have not explained the mechanisms behind the restoration of cartilage damage. The observed reduction in cartilage fibrillation and promotion of proteoglycan in histology, along with increased BMP2 expression due to curcumin nanoemulsion, suggests that BMP2 is involved in these processes. Bone morphogenetic protein-2, which is essential for bone and cartilage development, reduced cartilage damage and encouraged chondrocyte regeneration [56]. Typically, BMP2 transmits signals through the SMAD-dependent pathway [56]. The upregulation of BMP2 may result from the activation and phosphorylation of the BMP-specific receptor SMAD1, which was increased in this study. Furthermore, the role of BMP2 in the synthesis and maintenance of proteoglycans is well established [57]. Previous reports have shown that SMAD1 and SMAD5 have overlapping and interchangeable functions suggesting their redundant effects [58,59]. Hence, in the context of cartilage homeostasis, SMAD1 could still be involved in the BMP2 signaling cascade despite a decrease in SMAD5. Interestingly, overexpression of TGFβ has been linked to promoting an anti-inflammatory osteoarthritis (OA) microenvironment [60]. Curcumin nanoemulsion increased TGFβ expression in this study, emphasizing its role in both inflammation regulation and cartilage homeostasis. Curcumin nanoemulsion could promote ECM synthesis and chondrocyte proliferation via TGFβ [61]. A recent study showed that a deficiency of MMP14 expression led to inflammatory arthritis [62]. The increased expression of MMP14 and TIMP1 by curcumin nanoemulsion in this study may be part of a restorative mechanism reflecting either tissue or ECM remodeling or early fibrotic responses as observed by changes in the synovial membrane.

The reduced fibrillation of cartilage by curcumin nanoemulsion may also be due to its known anti-inflammatory activity. In line with several other studies [63,64,65,66], our results also show downregulation of NFkB and TNFα expression in chondrocytes. Previously, NFkB has been shown to regulate BMP2 expression, indicating its role in chondrogenesis [11]. NFkB is a key transcriptional regulator in synovial inflammation, modulating the expression of pro-inflammatory cytokines [9]. Curcumin has been shown to inhibit NFkB activity in osteoarthritis [63,66]. Upregulation of NFkB in OA-like macrophages was significantly reduced in the presence of curcumin nanoemulsion (Figure 6). Therefore, curcumin helps reduce the inflammatory environment associated with osteoarthritis by decreasing synovial inflammation, possibly by controlling macrophage recruitment through NF-κB inhibition.

Our study is limited to the fact that animal numbers in this study were significantly reduced due to animal stringency and regulatory concerns. A comparative assessment with native curcumin in vivo and further analysis of cartilage histology could not be achieved due to strict ethical obligation for providing novel data only using nanoemulsion, as the effects of curcumin are already established. However, the lack of comparative analysis with native curcumin is a limitation in this study that will be considered in future investigations. While the current findings with curcumin nanoemulsion suggest potential stability, comprehensive stability assessment, local safety profile, and drug release remain to be explored. Introduction of an additional time point during treatment might increase the translational value of nanoemulsion. Further, studying the impact of nanocurcumin on macrophage polarization in a cellular model would give better insights into its mechanism of action in addition to evaluation of functional parameters such as inflammatory cytokine release or proteoglycan synthesis.

## 4. Materials and Methods

### 4.1. Preparation of Nanoemulsion

Curcumin nanoemulsion was prepared as a micellar form using an oil-in-water approach by mixing 10 mM curcumin (#C7727, Sigma, St. Louis, MO, USA) with neutral triglyceride oil, Tween-20 (#P7949, Sigma) as surfactant, and deionized water. Oil and surfactant were taken in 1:1 ratio at ≤1% (*v*/*v*). The solution was sonicated at room temperature to aid emulsification and centrifuged. The prepared nanoemulsion was evaluated for its biophysical properties using a Zetasizer (Delsa^TM^ Nano, Beckman Coulter, Brea, CA, USA). Characterization was based on dynamic light scattering (nm) for size measurements, negative zeta potential for optical stability in solution, and polydispersity index (PDI) as a heterogeneity index of the particle size. A homogeneous micellar solution that fulfilled the biophysical characterization criteria (size < 200 nm; zeta potential < −30 mV; and PDI < 0.3%) was used in all subsequent experiments.

### 4.2. Animal Experiment

The animal study and involved protocols were conducted in accordance with the institutional guidelines and with the approval of the Institutional Animal Ethics Committee (#ICMR-NIN/IAEC/2024-I/002). In brief, 5-month-old male SD rats (n = 9) were procured from the animal facility, National Institute of Nutrition (NIN). Rats were housed individually in cages with a 12 h light/dark cycle at a temperature of 22 ± 2 °C and a relative humidity of 45–55%. Rats were given a standard chow diet (AIN93M) and water ad libitum. Sodium monoiodoacetate (MIA) (#I2512, Sigma, St. Louis, MO, USA) was used to induce osteoarthritis in rats according to the previously described protocol [67]. After one week of acclimatization, rats were randomly divided into three groups: control group (saline, vehicle), MIA-induced OA group (MIA, vehicle), and curcumin nanoemulsion group (MIA, treated with curcumin nanoemulsion, 30 mg/mL [68]). To induce osteoarthritis in rats, the left knee was shaved and cleaned with alcohol for disinfection. Except for the control group, a single dose of 2 mg MIA dissolved in 50 μL sterile saline was injected intra-articularly using a 31 G needle into the left knee. Rats in the control group were injected with sterile saline. After 4 weeks of disease induction using MIA rats, the rats in the curcumin nanoemulsion group received intra-articular injections of the nanoemulsion once a week for an additional 4 weeks, while the others were administered vehicle (sterile water). For administration of intra-articular injections rats were anesthetized using 3–5% isofluorane inhalation via a nose cone.

### 4.3. X-Ray Imaging and Scoring for Osteoarthritis

Radiographic images of the rat’s affected knee joint in anteroposterior and lateral views were obtained. Briefly, SD rats were anesthetized using ketamine (70 mg/kg)/xylazine (2.5 mg/kg) injection (intraperitoneal) and imaged under MRad 5.0 (BPL Medical Technologies, Bengaluru, India) using manual mode with phase voltage—240 V, kVp—70, mA—40, mAs—0.001, focal length—80 cm and developed using AGFA Musica. Images obtained were reviewed for OA-related changes following the Kellgren–Lawrence classification [69,70,71].

### 4.4. Knee Diameter Measurement

The diameter of the ipsilateral and contralateral knee joints was measured once a week throughout the study duration using digital vernier calipers (Mitutoyo, Kawasaki, Japan) to monitor MIA-induced edema. Measurements of knee diameter (mm) were taken in anteroposterior and mediolateral directions as described [72].

### 4.5. Collection of Blood and Knee Joints

Blood was collected in clot-activator vacutainers from rats at three time points via retro-orbital puncture, and the separated serum was stored at −80 °C for further analysis. An interval of 4 weeks was allowed between blood sample collection resulting in complete recovery of rats and blood was collected using micro hematocrit tubes under 3–5% isofluorane anesthesia. Rats were weighed before blood collection and not more than 10% of total blood volume (TBV) was collected assuming that TBV was roughly 7% of the rat’s body weight. All procedures were carried out only by trained personnel post the approval of the Institutional Animal Ethical Committee (IAEC). At the end of the experiment, rats were sacrificed by carbon dioxide asphyxiation. Intact knee joints were harvested and stored in 10% neutral buffered formalin for histopathology.

### 4.6. Serum Oxidative Stress

Oxidative stress in rats was assessed by measuring lipid peroxidation using the thiobarbituric acid reactive substances (TBARS) assay. Briefly, the serum was incubated with 0.3 M Tris-HCl buffer, 20% trichloroacetic acid (#Q28444, Qualigens, Thermo Fisher, Mumbai, India), and 0.67% thiobarbituric acid (#T5500-25G, Sigma). Then, it was boiled and centrifuged to precipitate the protein. The supernatant was used to measure absorbance at 532 nm against malonaldehyde (MDA) (#108383, Sigma) as the standard using a 96-well microplate reader (Biotek, PowerWave XS, Winooski, VT, USA).

### 4.7. Knee Joint Histology and Staining

Left knee joints fixed in 10% formalin were decalcified using 5% formic acid for 2 weeks and embedded in paraffin as described previously [73]. Specimen blocks were cut into 4 μm sections. The sections were deparaffinized, rehydrated, and stained using Haematoxylin and Eosin (H&E). Additionally, knee joint sections were also stained using safranin O (#16593, SRL, Mumbai, India) or toluidine blue (#22134, SRL) and counter-stained using fast green (#60339, SRL) as previously described [74]. The sections were visualized and imaged under a light microscope at 10x and 40x magnification (Eclipse TE2000U, Nikon, Tokyo, Japan). The histopathological changes were determined quantitatively using the modified Mankin scoring system [75,76] by three independent blinded observers on multiple field of views per group based on the criteria given in Table S3.

### 4.8. Reagents and Cell Culture

CHON-001 chondrocyte cell line (#CRL_2846) and RAW 264.7 macrophage cell line (#TIB-71) were obtained from the American Type Culture Collection (ATCC), (Manassas, VA, USA). IL1β (#201-LB-005) was procured from R&D Systems (Minneapolis, MN, USA). Fetal Bovine Serum (FBS) (#10270106), Trypsin-EDTA (#25300062), and penicillin/streptomycin solution (#15140122) were obtained from Thermo Fischer Scientific, (Waltham, MA, USA). Dulbecco’s Modified Eagle Medium/High Glucose (DMEM) (#SH30249.01) was acquired from Cytiva, HyClone laboratories (Logan, UT, USA). F12 nutrient-rich medium (#21127-022) was obtained from Gibco (Thermo Fisher, Waltham, MA, USA). Curcumin (#C7727), Methyl thiazolyl diphenyl-tetrazolium bromide (MTT) (#5655), and Dimethyl sulfoxide (DMSO) (#D2650) were procured from Sigma Aldrich (St. Louis, MO, USA).

CHON-001 cells were cultured in DMEM/F12 supplemented with 10% heat-inactivated FBS and 1% Penicillin/Streptomycin. RAW 264.7 cells were maintained in DMEM supplemented with 10% FBS and 1% antibiotics (penicillin/streptomycin). Cells were maintained at 37 °C in a 5% CO_2_ incubator. The cells were seeded in 6-well plates and serum-starved for 6 h, followed by incubation with IL1β (10 ng/mL) for 24 h to induce OA-like cells. Curcumin was dissolved in DMSO to prepare stock solution and further diluted with assay media keeping DMSO at 0.01% (*v*/*v*). Nanoemulsion was prepared as described earlier. Curcumin or its nanoemulsion (5 μM,10 μM) were administered to cells in the presence of IL1β.

### 4.9. Cell Viability by MTT Assay

Cells were seeded at a density of 1 × 10^4^ cells in 96-well plates and serum-starved for 6 h. Following this, the cells were exposed to different concentrations of native curcumin and its nanoemulsion. After 24 h, the assay media were removed, and MTT (5 mg/mL) was added. The cells were then incubated for an additional 4 h to generate formazan crystals. The resulting crystals were solubilized with DMSO (100 μL), and the absorbance of the samples was determined using a microplate reader at 562 nm (BioTek, PowerWave XS, Winooski, VT, USA).

### 4.10. Immunoblotting

Immunoblotting in cell lysates was performed as described previously [77] to assess protein expression. The nitrocellulose membranes (#1620112, Bio-Rad, Hercules, CA, USA) were incubated with primary antibody NF-κB (#SC-8008, Santa Cruz Biotechnology, Dallas, TX, USA) at a 1:1000 dilution. HRP-conjugated anti-mouse antibody (#31430, Invitrogen, Thermo Fisher, Carlsbad, CA, USA) was used for secondary incubation, followed by chemiluminescent imaging (iBright FL1500, Invitrogen). The blots were quantified and analyzed using ImageJ software, version 1.50i (NIH, Bethesda, MD, USA).

### 4.11. Quantitative Real-Time PCR

RNA isolation was performed from chondrocytes (CHON-001) using TRIzol (#T9424, Sigma-Aldrich). cDNA synthesis and quantitative real-time PCR were performed using a CFX-96 well real-time PCR system (Bio-Rad, USA) as previously described [77]. KiCqstart primers (Sigma) were used as mentioned (Table S5). A panel of candidate housekeeping genes was assessed, and stability values were derived using the NormFinder algorithm to identify the optimal endogenous control (Table S6). Relative quantification of gene expression was calculated using ddCt method.

### 4.12. Detection of NFκB Localization by Immunocytochemistry

Cells cultured on coverslips were fixed using 4% paraformaldehyde for 20 min. After permeabilization with 0.25% Triton-X 100 (#RM845, Himedia, Mumbai, India) and being rinsed with phosphate-buffered saline (PBS), cells were blocked with 5% BSA and incubated with primary antibody NF-κB (#SC-8008, SantaCruz Biotechnology, Dallas, TX, USA) with a dilution of 1:250 at 4 °C overnight. The next day, cells were incubated with secondary antibody conjugated with AlexaFluor 594 (#A11032, Invitrogen, Thermo Fisher, Carlsbad, CA, USA) for 2 h after rinsing with PBS. The nuclei were counter-stained, and coverslips were mounted onto slides with fluoromount containing DAPI (#ab104139, Abcam). Slides were imaged using a confocal microscope (TCS SP5, Leica Microsystems, Wetzlar, Germany) at 40× magnification.

### 4.13. Transwell Co-Culture Chemotaxis Assay

Mimicking the OA microenvironment, this study employed a co-culture model of chondrocyte (CHON 001) and macrophage (RAW 264.7) to evaluate the chemotactic effect of IL1β-stimulated chondrocyte-derived factors on the migration of macrophages. Chondrocytes were stimulated with IL1β to mimic osteoarthritis-related inflammatory responses in vitro and treated with curcumin or nanoemulsion for comparative evaluation. Chemotactic migration of macrophages was performed using a previously described protocol with slight modifications [19]. Briefly, CHON-001 cells (1 × 10^5^) were seeded in the lower chamber of a 24-well plate, serum-starved, and treated with curcumin or curcumin nanoemulsion (10 μM) in the presence of IL1β (10 ng/mL) for 24 h. After 24 h, serum-starved RAW 264.7 macrophages (2 × 10^4^) were added to the transwell hanging inserts (#37224, SPL Life Sciences, Pocheon-si, South Korea) with 8.0 μm pore size. The inserts were positioned into the wells containing stimulated chondrocytes and incubated for another 24 h. Spent media was aspirated, and non-migratory macrophages from the apical side of the transwell insert were gently removed using a swab. Macrophages on the underside of the insert membrane were stained with 5 μM calcein AM dye (#C1430, Invitrogen, Thermo Fisher, Carlsbad, CA, USA) for 30 min and visualized using a fluorescent inverted microscope, 10× magnification (Eclipse TE2000U, Nikon, Tokyo, Japan). Mean migrated cells were analyzed using three random fields per image [78].

### 4.14. Statistical Analysis

Statistical analysis was performed with the GraphPad Prism v.8 platform. Each experiment was conducted independently and replicated multiple times, as described in the text or figure legends, with values being represented as mean ± SEM. Analysis of repeated measurements was carried out using a repeated-measures mixed-effects ANOVA, parametric one-way ANOVA with post hoc Tukey’s test for multiple comparisons or Kruskal–Wallis test with Dunn’s multiple comparisons for non-parametric data was used and *p* < 0.05 was considered statistically significant, with unlike letters representing a significant difference.

## 5. Conclusions

Overall, the present study shows that the localized delivery of dietary oil formulated curcumin nanoemulsion displayed comprehensive anti-inflammatory effects in the osteoarthritis model. This is the first comprehensive study demonstrating the effect of curcumin nanoemulsion on chondrocyte–macrophage interactions in the OA microenvironment. In particular, curcumin mitigated cartilage degeneration by reducing fibrillation, hypocellularity, and restoring staining of proteoglycans, which could be due to reduced expression of NFkB in chondrocytes. Curcumin nanoemulsion also reduced synovial inflammation, potentially by suppression of NFkB in macrophages, and mitigated macrophage recruitment indicating its anti-inflammatory role. These inhibitory effects of curcumin nanoemulsion suggest its potential as an NFkB-suppressing therapeutic compound.

## Figures and Tables

**Table 1:** Relative mRNA expression ^1^ of pro-inflammatory and matrix regulatory genes in chondrocytes after treatment with curcumin nanoemulsion ^2^.

Type of Gene	Gene Symbol	Control (Unstimulated)	IL1β (10 ng/mL)	IL1β + Cur (10 μM)	IL1β + Cur-Nanoemulsion (10 μM)
**Inflammatory Modulators**					
	NFκB	1.01 ± 0.04 ^a^	4.41 ± 0.67 ^b^	2.37 ± 0.05 ^c^	2.47 ± 0.07 ^c^
	NFκB-IB	1.01 ± 0.06 ^a^	2.28 ± 0.32 ^b^	3.14 ± 0.33 ^b^	3.58 ± 0.22 ^bc^
	TNFα	1.01 ± 0.04 ^a^	1.65 ± 0.11 ^b^	0.90 ± 0.03 ^a^	0.97 ± 0.09 ^a^
**Matrix Regulators**					
	TGFβ1	1.01 ± 0.02 ^a^	0.57 ± 0.03 ^b^	1.35 ± 0.06 ^a^	1.24 ± 0.17 ^a^
	BMP2	1.01 ± 0.05 ^a^	8.61 ± 2.29 ^b^	12.78 ± 1.07 ^b^	13.64 ± 1.31 ^b^
	SMAD1	1.01 ± 0.04 ^a^	0.95 ± 0.05 ^a^	2.20 ± 0.07 ^b^	1.40 ± 0.08 ^c^
	SMAD5	1.01 ± 0.02 ^a^	0.70 ± 0.22 ^a^	0.52 ± 0.02 ^a^	0.32 ± 0.02 ^ab^
	SPARC	1.01 ± 0.02 ^a^	0.39 ± 0.13 ^b^	0.13 ± 0.01 ^b^	0.18 ± 0.01 ^b^
	MMP2	1.01 ± 0.01 ^a^	1.57 ± 0.10 ^b^	1.56 ± 0.07 ^b^	1.44 ± 0.18 ^b^
	MMP9	1.01 ± 0.02 ^a^	1.94 ± 0.49 ^b^	0.93 ± 0.16 ^a^	1.94 ± 0.13 ^b^
	MMP14	1.01 ± 0.02 ^a^	0.84 ± 0.23 ^a^	1.52 ± 0.09 ^b^	1.47 ± 0.04 ^ab^
	TIMP1	1.01 ± 0.03 ^a^	1.98 ± 0.32 ^b^	2.08 ± 0.18 ^b^	2.53 ± 0.16 ^b^

^1^ The level of mRNA expression of genes was quantified after normalizing with endogenous control, TBP, and calculated according to the 2^−ΔΔCt^ method. Data are expressed as mean of relative mRNA ± SEM, n = 3. ^2^ mRNA expression was measured in IL1β-stimulated CHON-001 cells after incubating with curcumin (10µM) or curcumin nanoemulsion (10µM) 24 h. Unlike superscript letters denote significance with *p* < 0.05 vs. control.

## Data Availability

The original contributions presented in this study are included in the article/Supplementary Materials. Further inquiries can be directed to the corresponding author.

## Supplementary Materials

The following supporting information can be downloaded at: https://www.mdpi.com/article/10.3390/ijms262211212/s1.
