# Correction: Hridayanka et al. Intra-Articular Delivery of Nanoemulsified Curcumin Ameliorates Joint Degeneration in a Chemically Induced Model of Osteoarthritis. *Int. J. Mol. Sci.* 2025, *26*, 11212

**DOI:** 10.3390/ijms27156985

**Published:** 2026-08-04

**Authors:** Kota Sri Naga Hridayanka, Shibsekhar Roy, Saikanth Varma, Navya Sree Boga, Archana Molangiri, Pradeep B. Patil, Myadara Srinivas, Asim K. Duttaroy, Sanjay Basak

**Affiliations:** 1 Molecular Biology Division, National Institute of Nutrition, Indian Council of Medical Research, Hyderabad 500007, Indianavyasreeboga.nice@gmail.com (N.S.B.);; 2 Department of Biochemistry, University College of Science, Osmania University, Hyderabad 500007, India; 3 Division of Animal Facility, National Institute of Nutrition, Indian Council of Medical Research, Hyderabad 500007, India; 4 Department of Nutrition, Institute of Basic Medical Sciences, Faculty of Medicine, University of Oslo, 0316 Oslo, Norway; a.k.duttaroy@medisin.uio.no

**Text Correction**

A correction has been made to paragraph 3 of the Discussion section: 

The sentence and its corresponding citation ‘{Curcumin may also modulate alterations in the subchondral bone by suppressing osteoclastogenesis [42]}’ have been deleted from the original publication [1] following the retraction of the cited source.

42. Ding, D.; Liu, G.; Yan, J.; Zhang, Q.; Meng, F.; Wang, L. Curcumin alleviates osteoarthritis in mice by suppressing osteoclastogenesis in subchondral bone via inhibiting NF-κB/JNK signaling pathway. *PLoS ONE* **2024**, *19*, e0309807.

The authors state that the scientific conclusions are unaffected. This correction was approved by the Academic Editor. The original publication has also been updated.
