# Supplementary material for: Intra-Articular Delivery of Nanoemulsified Curcumin Ameliorates Joint Degeneration in a Chemically Induced Model of Osteoarthritis
Source: Int J Mol Sci. 2025 Nov 20;26(22):11212. doi: 10.3390/ijms262211212 (PMC12653435; doi:10.3390/ijms262211212)
Supplement: Supplementary file 1 [file ijms-26-11212-s001.zip › Supplementary Table S1.pdf]

Table S1: Biophysical properties of curcumin nanoemulsion[1]

Parameter	Value 1	Value 2
Particle size (nm)	184.29	209.60
Zeta Potential (mV)	-34.00	-32.60
Polydispersity Index (%)	31.20	28.50

[1] characterization of curcumin nanoemulsion was carried out using Zetasizer (Delsa™ Nano, Beckman Coulter, USA).
