# Supplementary material for: Intra-Articular Delivery of Nanoemulsified Curcumin Ameliorates Joint Degeneration in a Chemically Induced Model of Osteoarthritis
Source: Int J Mol Sci. 2025 Nov 20;26(22):11212. doi: 10.3390/ijms262211212 (PMC12653435; doi:10.3390/ijms262211212)
Supplement: Supplementary file 1 [file ijms-26-11212-s001.zip › Supplementary Table S2.pdf]

Table S2: Grading of X-ray radiographs of the knee joints of rats based on Kellgren-Lawrence classification [1]

Parameter	Grade
No joint space narrowing or reactive changes	0
Doubtful joint space narrowing, possible osteophytic lipping	1
Definite osteophytes, possible joint space narrowing	2
Moderate osteophytes, definite joint space narrowing, some sclerosis, possible bone-end deformity	3
Large osteophytes, marked joint space narrowing, severe sclerosis, definite bone-ends deformity	4

1. Kohn, M.D.; Sassoon, A.A.; Fernando, N.D. Classifications in Brief: Kellgren-Lawrence Classification of Osteoarthritis. *Clinical orthopaedics and related research* **2016**, *474*, 1886-1893, doi:10.1007/s11999-016-4732-4.
