# Supplementary material for: Intra-Articular Delivery of Nanoemulsified Curcumin Ameliorates Joint Degeneration in a Chemically Induced Model of Osteoarthritis
Source: Int J Mol Sci. 2025 Nov 20;26(22):11212. doi: 10.3390/ijms262211212 (PMC12653435; doi:10.3390/ijms262211212)
Supplement: Supplementary file 1 [file ijms-26-11212-s001.zip › Supplementary Table S3.pdf]

Table S3: Modified Mankin scoring [1,2] for osteoarthritis based on histological findings in the knee joints of rats

	Histological findings	Score
**Structure**		
	Normal	0
	Surface irregularities	1
	Pannus and surface irregularities	2
	Clefts to transitional zone	3
	Clefts to radial zone	4
	Clefts to calcified zone	5
	Complete disorganization	6
**Cellularity**		
	Normal	0
	Increase or slight decrease	1
	Moderate decrease	2
	Severe decrease	3
	No cells	4
**Safranin O Staining**		
	Normal	0
	Slight decrease	1
	Moderate decrease	2
	Severe decrease	3
	No staining	4
**Tidemark Integrity**		
	Normal	0
	Disappearance or invasion by vessels	1

1. Mankin, H.J.; Dorfman, H.; Lippiello, L.; Zarins, A. Biochemical and metabolic abnormalities in articular cartilage from osteo-arthritic human hips. II. Correlation of morphology with biochemical and metabolic data. *The Journal of bone and joint surgery. American volume* **1971**, *53*, 523-537.
2. Takahashi, I.; Matsuzaki, T.; Kuroki, H.; Hoso, M. Joint unloading inhibits articular cartilage degeneration in knee joints of a monosodium iodoacetate-induced rat model of osteoarthritis. *Osteoarthritis and cartilage* **2019**, *27*, 1084-1093, doi:10.1016/j.joca.2019.03.001.
