# Supplementary material for: Intra-Articular Delivery of Nanoemulsified Curcumin Ameliorates Joint Degeneration in a Chemically Induced Model of Osteoarthritis
Source: Int J Mol Sci. 2025 Nov 20;26(22):11212. doi: 10.3390/ijms262211212 (PMC12653435; doi:10.3390/ijms262211212)
Supplement: Supplementary file 1 [file ijms-26-11212-s001.zip › Supplementary Table S4.pdf]

Table S4: Scoring of synovial membrane inflammation based on histological findings in the knee joints of rats [1]

Synovial membrane inflammation	Score
No changes (1-2 layers of synovial cells)	0
Increased number of lining cell layers (≥ 3-4 cell layers) or slight proliferation of subsynovial tissue	1
Increased number of lining cell layers (≥ 3-4 cell layers) and/or proliferation of subsynovial tissue	2
Increased number of lining cell layers (>4 layers) and/or proliferation of subsynovial tissue and infiltration of few inflammatory cells	3
Increased number of lining cell layers (>4 layers) and/or proliferation of subsynovial tissue and infiltration of large number of inflammatory cells	4

1. Gerwin, N.; Bendele, A.M.; Glasson, S.; Carlson, C.S. The OARSI histopathology initiative - recommendations for histological assessments of osteoarthritis in the rat. *Osteoarthritis and cartilage* **2010**, *18 Suppl 3*, S24-34, doi:10.1016/j.joca.2010.05.030.
