# Supplementary material for: Intra-Articular Delivery of Nanoemulsified Curcumin Ameliorates Joint Degeneration in a Chemically Induced Model of Osteoarthritis
Source: Int J Mol Sci. 2025 Nov 20;26(22):11212. doi: 10.3390/ijms262211212 (PMC12653435; doi:10.3390/ijms262211212)
Supplement: Supplementary file 1 [file ijms-26-11212-s001.zip › Supplementary Table S5.pdf]

Table S5: List of predesigned KiCqStart® SYBR® Green I pre-designed primers for gene expression analysis

Sl. No.	Primer ID	Gene symbol	Gene ID	Gene name	Nucleotide sequences (5'-3')	Ref_seq ID
1	H_NFKB1_1	NFKB1	4790	Nuclear factor kappa B subunit 1	F 5'- CACAAGGAGACATGAAACAG-3'<br>R 5'- CCCAGAGACCTCATAGTTG-3'	NM_003998
2	H_NFKBIB_1	NFKBIB	4793	Nuclear factor kappa B inhibitor beta	F 5'- CGATGAATACGACGACATTG-3'<br>R 5'- CAATGAAACAAATCACACGG-3'	NM_002503
3	H_TGFB1_3	TGFB1	7040	Transforming growth factors-1	F 5'- TGTACCAGAAATACAGCAAC-3'<br>R 5'- CAAAAGATAACCACTCTGGC-3'	NM_000660
4	H_TNFA_1	TNFA	7124	Tumor necrosis factor	F 5'- CCTCTCTCTAATCAGCCCTCTG-3'<br>R 5'- GAGGACCTGGGAGTAGATGAG-3'	NM_00594
5	H_MMP2_3	MMP2	4313	Matrix metalloproteinases 2 /type IV collagen	F 5'- ATGAATACTGGATCTACTCAGC-3'<br>R 5'- GTATCTCCAGAATTTGTCTCC-3'	NM_001127891
6	H_MMP9_3	MMP9	4318	Matrix metalloproteinases 9/type IV collagen	F 5'- CTTAGATCATTCCTCAGTGC-3'<br>R 5'- CGAGGACCATAGAGGTG-3'	NM_004994
7	H_MMP14_1	MMP14	4323	Matrix metalloproteinases 14	F 5'- ATGGCAAATTCGTCTTCTTC-3'<br>F 5'- CGTTGAAACGGTAGTACTTG-3'	NM_004995
8	H_TIMP1_1	TIMP1	7076	Tissue inhibitor metalloproteinases 1	F 5'- CACCTTATACCAGCGTTATG-3'<br>R 5'- TTTCCAGCAATGAGAAACTC-3'	NM_003254
9	H_SPARC_1	SPARC	6678	Osteonectin	F 5'- AGTATGTGTAACAGGAGGAC-3'<br>R 5'- AATGTTGCTAGTGTGATTGG-3'	NM_003118
10	H_BMP2_1	BMP2	650	Bone morphogenic protein 2	F 5'- TCCACCATGAAGAATCTTTG-3'<br>R 5'- TAATTCGGTGATGGAAACTG-3'	NM_001200
11	H_SMAD1_1	SMAD1	4086	Sma and Mad related proteins - 1	F 5'- GGCATATTGGAAAAGGAGTTC-3'<br>R 5'- AGATGCTACTGTCACTAAGG-3'	NM_005900
12	H_SMAD5_1	SMAD5	4090	Sma and Mad related proteins - 5	F 5'- CCAGTCTTACCTCCAGTATTAG-3'<br>R 5'- TCCTAAACTGAACCAGAAGG-3'	NM_005903
13	H_TBP_1	TBP	6908	TATA binding protein	F 5'- GCCAAGAGTGAAGAACAG-3'<br>R 5'- GAAGTCCAAGAACTTAGCTG-3'	NM_003194
14	H_B2M_1	B2M	567	Beta-2-microglobulin	F 5'- AAGGACTGGTCTTTCTATCTC-3'<br>R 5'- GATCCCACTTAACTATCTGG-3'	NM_004048
15	H_GAPDH_1	GAPDH	2597	Glyceraldehyde-3-phosphate dehydrogenase	F 5'- TCGGAGTCAACGGATTTG-3'<br>R 5'- CAACAATATCCACTTTACCAGAG-3'	NM_002046
16	H_ACTB_1	ACTB	60	Beta actin	F 5'- GACGACATGGAGAAAATCTG-3'<br>R 5'- ATGATCTGGGTCATCTTCTC-3'	NM_001101
17	H_YWHAZ_1	YWHAZ	7534	Tyrosine 3-monooxygenase/tryptophan 5-monooxygenase activation protein zeta	F 5'- AACTTGACATTGTGGACATC-3'<br>R 5'- AAAACTATTTGTGGGACAGC-3'	NM_003406
