# Supplementary material for: Intra-Articular Delivery of Nanoemulsified Curcumin Ameliorates Joint Degeneration in a Chemically Induced Model of Osteoarthritis
Source: Int J Mol Sci. 2025 Nov 20;26(22):11212. doi: 10.3390/ijms262211212 (PMC12653435; doi:10.3390/ijms262211212)
Supplement: Supplementary file 1 [file ijms-26-11212-s001.zip › Supplementary Table S6.pdf]

Table S6: Stability assessment of panel of candidate housekeeping genes analysed using NormFinder[1]

**Gene name**	**Stability value**
GAPDH	0.021
B2M	0.039
TBP	0.002
BACTIN	0.024
YWHAZ	0.015
**Best gene**	TBP
**Stability value**	0.002
**Best combination of two genes**	TBP and YWHAZ
**Stability value for best combination of two genes**	0.009

[1] Gene with the lowest value indicates the highest stability (TBP – 0.002 stability value)
