# Supplementary figures and images for: Intra-Articular Delivery of Nanoemulsified Curcumin Ameliorates Joint Degeneration in a Chemically Induced Model of Osteoarthritis
Source: Int J Mol Sci. 2025 Nov 20;26(22):11212. doi: 10.3390/ijms262211212 (PMC12653435; doi:10.3390/ijms262211212)

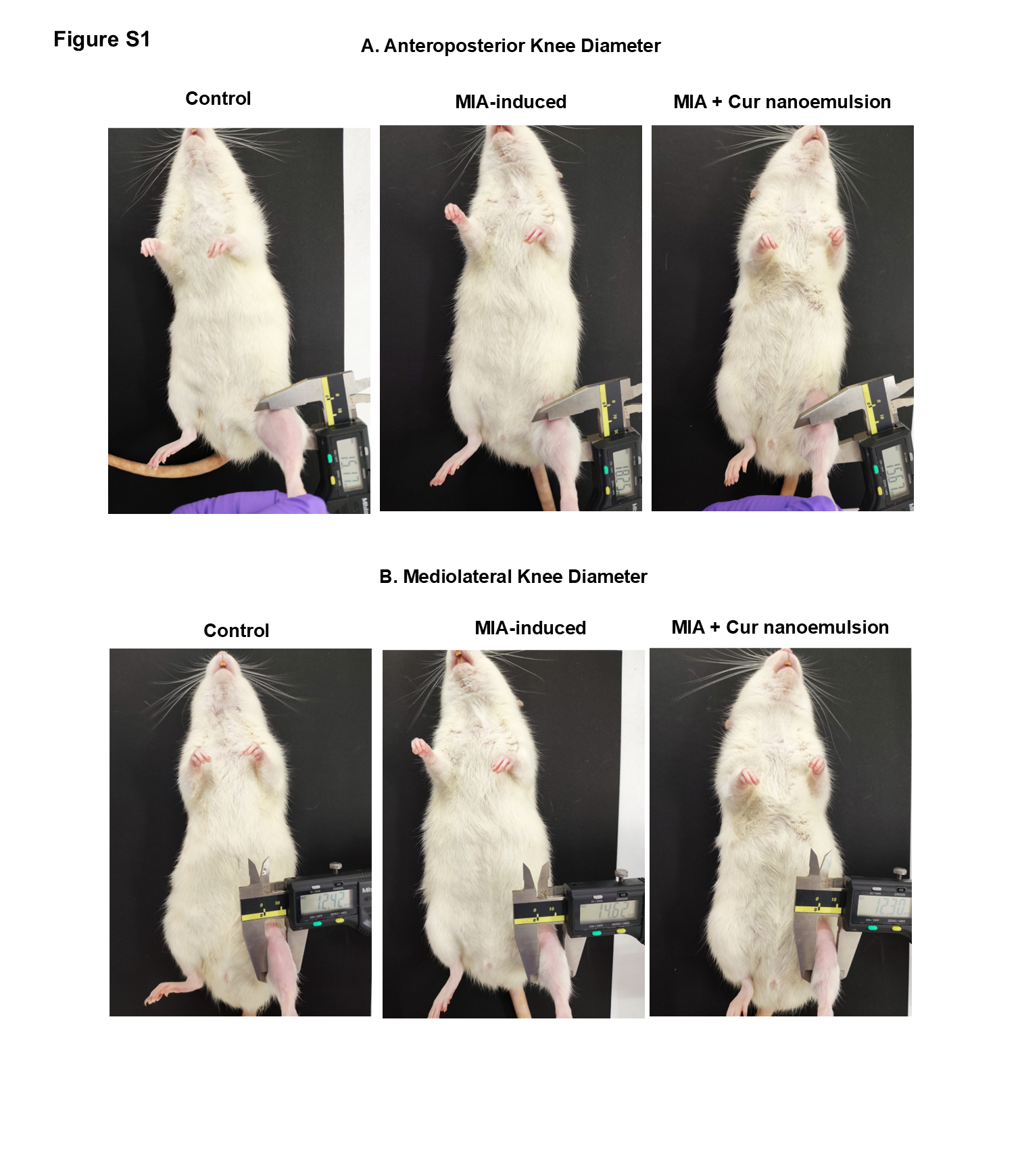

Supplement: Supplementary file 1 [file ijms-26-11212-s001.zip › Supplementary Figure S1.TIF]
